# Application of Poly(lactic Acid) Composites in the Automotive Sector: A Critical Review

**DOI:** 10.3390/polym16213059

**Published:** 2024-10-30

**Authors:** Valentina Giammaria, Monica Capretti, Giulia Del Bianco, Simonetta Boria, Carlo Santulli

**Affiliations:** 1 School of Science and Technology, Mathematics Division, University of Camerino, Via Madonna delle Carceri 9, 62032 Camerino, Italy; valentina.giammaria@unicam.it (V.G.); monica.capretti@unicam.it (M.C.); giulia.delbianco@unicam.it (G.D.B.); simonetta.boria@unicam.it (S.B.); 2 School of Science and Technology, Geology Division, University of Camerino, Via Gentile III da Varano 7, 62032 Camerino, Italy

**Keywords:** bio-based polymers, natural fibers, mechanical characteristics, additive manufacturing (AM), vehicle components

## Abstract

The introduction of bio-based matrices in automotive applications would, in principle, increase their sustainability and, in case the use of secondary raw materials is also involved, even result in reduced resource depletion. The bio-based polymer composite matrix that has been mainly brought forward towards industrial application is poly(lactic acid) (PLA), which has often been proposed as the replacement for matrices based on polyolefins in fields such as packaging and short-term commodities since, in general, it matches the needs for conventional thermoplastic production processes. The passage to the automotive sector is not obvious, though: problems affecting durability, the relation with water and the environment, together with the requirement for outstanding mechanical and impact performance appear very stringent. On the other hand, PLA has obtained durable success in additive manufacturing as a competitor for acrylonitrile butadiene styrene (ABS). Also, the perspective for 3D and 4D printing does not appear to be confined to bare prototyping. These contrasting pieces of evidence indicate the necessity to provide more insight into the possible development of PLA use in the automotive industry, also considering the pressure for the combined use of more sustainable reinforcement types in automotive composites, such as natural fibers.

## 1. Introduction

The penetration of bio-based polymers in the automotive industry is continuously increasing, with the idea to reduce the reliance on petrochemical products in vehicle production [1]. Most recently, the penalty in terms of performance and resins using bio-based ones has been constantly reduced and even reversed by providing chemical functionalities to the level of traditional polymers [2]. Dealing with biopolymers, whose application has been proposed in the automotive industry with particular drive in the last decade [3], the principal possible routes are: (i) the modification of partially bio-based polymers, such as starch, into a bioplastic [4]; (ii) the obtainment of a bio-based monomer, such as lactic acid, to repolymerize at different molecular weights; (iii) the direct production of biopolymers through micro-organisms, such as bacteria [5]. Respective categories of products that are obtained are: (i) thermoplastic starches (TPS) [6]; (ii) bio-based thermoplastic polyesters, such as poly(lactic acid) (PLA) [7]; (iii) bacterial bioplastics, collectively indicated as poly(hydroxyalkanoates) (PHAs), among which the most diffuse polymer is poly(hydroxybutyrate) (PHB) [8]. In this context, PLA, for its more controllable properties and relatively cheap price, does appear the most suitable candidate for the substitution of (or blending with) oil-based thermoplastics, such as poly(ethylene) (PE) and polypropylene (PP) in contexts such as the automotive industry [9]. Other possible competitors with PLA can be bio-based PE, PP, or poly(ethylene terephthalate) (PET), obtained from the treatment of agricultural ethanol [10]. The introduction of PLA in general, and more specifically in the automotive industry, leads to two other significant consequences: the wider penetration of natural (lignocellulosic) fibers as reinforcement for composites [11] and the exploitation of the potential of the additive manufacturing process (3D printing) for structural, yet at least partially bio-based, components [12]. This work explores the potential of PLA in this sector, drawing on the most recent developments in PLA composites in the specific field of the automotive industry, where the declining production trends and the need for a lower footprint in the various phases of operation suggest the paramount importance of material innovation. To the concept level, PLA composites have been suggested to be ready for various applications in the vehicle, among which are wheel boxes, boot linings, door panels, storage and noise insulation panels [13]. However, to really enter the assembly line, this material needs other than impact, creep, and fatigue studies, which are at the forefront in this sector, also to offer other characteristics, among which is fire retardancy, which is intensely investigated for PLA [14]. These issues, together with the thriving market of natural fiber reinforcements, with new types of species and textile products being continuously developed [15], suggest critically taking stock of the evolution of the perspectives of PLA composites in the automotive industry.

## 2. The Use of PLA in Composites

The growing emphasis on flexibility and ease of manufacturing has led industries to seek innovative solutions aimed at minimizing costs and waste while still preserving design flexibility and material efficiency [16]. For this reason, additive manufacturing (AM) is widely applied in aircraft, construction, and automotive industries for rapid prototyping. Despite these interesting advantages, it is necessary to observe that the mechanical properties of printed samples are very low and, therefore, not suitable for structural applications [17]. To overcome this issue, researchers have explored different solutions obtained by integrating PLA with synthetic, and natural fibers, creating composites. Therefore, this section is focused on the existing literature regarding PLA composites: the discussion starts with the analysis of carbon, glass, and kevlar as the most promising reinforcements for automotive components. Then, the focus moves towards purely natural fibers; in particular, flax, jute, and kenaf/PLA composites are discussed in detail.

### 2.1. PLA in Synthetic and Hybrid Composites

When talking about synthetic fibers, one cannot avoid mentioning carbon. Due to its excellent mechanical properties, it is suitable for numerous applications, especially in the automotive industry. The addition of carbon (C) fibers to PLA significantly improves its properties. However, such an improvement is not so obvious: just think about the presence of short carbon fibers (SCFs), which do not allow a significant increase in resistance compared to the properties of pure PLA. To emphasize this aspect, reference can be made to Ferreira et al. [18] and Alkabbanie et al. [19], who investigated the tensile, flexural, shear, and impact properties of SCF/PLA composites. A fiber content of 15% is taken into account in both these studies. Compared to pure PLA, the addition of SCFs enables an increase in the elastic and shear moduli only in the printing direction. On the contrary, the tensile and shear stiffnesses are more or less the same, revealing that the presence of SCFs does not affect these properties. The flexural strength of SCF/PLA composites increases by almost 24% compared to pure PLA. In contrast, a significant reduction in impact strength can be observed: 38.45 and 77.47 kJ/m^2^ are obtained for SCF/PLA and pure PLA, respectively. All these results indicate that the introduction of SCFs in PLA leads to a complex interplay of mechanical properties. Moreover, the limited length of the fibers (∼60 µm) reduces the ability of the material to absorb energy and does not guarantee good fiber-matrix adhesion due to the small contact area. There is also a greater likelihood of irregularities and stress concentrations developing, which promote the formation and propagation of cracks and reduce the strength of the composites [19]. These limitations make SCF composites unsuitable for industrial high-performance applications; therefore, researchers look for the development of continuous carbon fiber (CCF)/PLA composites. As evidence, the work of Maqsood et al. [20] is proposed, in which the tensile and flexural properties of SCF/PLA and CCF/PLA composites are compared, taking into account a carbon content of 18%. All values are listed in Table 1, which shows that the tensile strength of composites made with CCFs is about six times higher than that of SCFs. The bending stress also increases significantly: compared to SCF/PLA, there is an increase of 121%. An opposite trend can be seen for the maximum tensile strain: it decreases in the case of CCFs.

A way to improve the mechanical properties of CCF/PLA printed composites is to increase the fiber volume fraction (FVF). In their work, Uşun et al. [21] discussed the effect of CCFs on tensile and flexural properties of PLA-based printed composites, considering a FVF up to 40%. To provide an easier comparison, all the obtained results are collected in Table 2. A look at the table reveals that performance is enhanced with an increase in the FVF: as it rises from 22 to 40%, the tensile strength value increases by 60.5%, whereas the flexural strength exhibits a lower increase equal to 21%.

Despite the better mechanical properties, an increase in the percentage of voids is observed. In particular, a porosity fraction of 11.9, 9.3, and 6.4% is measured for FVF of 40, 33, and 22%, respectively. The increase in porosity fraction has a negative impact on performance; consequently, higher strength loss has been observed in specimens with a higher fiber fraction when filament and printed parts are compared. Another aspect that is worth mentioning is the high influence given using different parameters, such as layer thickness, fiber orientation, printing temperature, and speed. This aspect can be demonstrated by comparing the results provided by Chen et al. [22] with those already discussed. In particular, using CCF/PLA samples with 33% of FVF, a tensile strength of 248.90 MPa is obtained, revealing a reduction of almost 75% with respect to the strength obtained in [21] using the same FVF. A significant reduction is also observed for the bending strength, for which a value of 104.20 MPa is achieved. This suggests that, even if the same printing technique is used, the obtained composites are very sensitive to production process parameters and, therefore, it is essential for researchers to investigate the optimization of these criteria.

In addition to carbon, glass (G) fibers are also widely used for high-performance composites. In the work of Varsavas et al. [23], the mechanical properties of G/PLA composites are analyzed. 5, 10, 15, and 20% fiber content are investigated, and a positive correlation between performance and glass content is found. For example, at a fiber content of 20%, the tensile and flexural strength increase by 30 and 7%, respectively, if compared to pure PLA. Moreover, the elastic and flexural moduli increase significantly by 53 and 121%, respectively. However, many voids and fiber pull-outs are observed at the interface. To overcome this problem and improve their applicability, some surface modifications are proposed in the literature [24,25].

As is common for composites with thermoset matrices, the combination of different fiber types can be considered a valid solution to improve the composite performance. An example of this are hybrid samples obtained by mixing 16% glass with carbon fiber, which corresponds to a total volume fraction of 49% [22]. The tensile and flexural properties are discussed and compared to those obtained when using pure carbon. The maximum tensile strength for the hybrids is 282.40 MPa and the elastic modulus is 10.90 GPa; thus, an increase of almost 14% is observed compared to the CCF/PLA samples, indicating that the synergistic effect improves the tensile strength behavior. An opposite trend can be observed for the flexural properties: a reduction of 37 and 75% is obtained for the stress and modulus, respectively. This difference is attributed to the presence of tiny gaps at the junctions of the two fibers, during the impregnation process. Therefore, the interface contact between single-fiber layers appears to be more effective than that between layers of hybrid fibers. However, the promising results described so far suggest the need to investigate further hybrid solutions that can lead to improve the performance. Other interesting results are those proposed by Yang et al. [26], who study the behavior of kevlar (K) and ramie–kevlar/PLA composite, demonstrating the good effects coming from the combination of both synthetic and natural fibers. All the considered composites consist of 5 layers, and the stacking sequence for the hybrid case is the following: KKRKK, where K stands for kevlar and R for ramie. The tensile strength of 5K/PLA composite is equal to 250 MPa and, therefore, can be considered sufficiently high to be competitive with low-strength metals. Moreover, a reduction of only 4% is observed if one kevlar layer is substituted by ramie. Regarding instead the flexural strength, values of 182 and 208 MPa are obtained for kevlar and hybrid composites, respectively. The presence of ramie fibers in the inner part benefits the bending resistance because of the better fiber-matrix interfacial adhesion given by ramie with respect to kevlar.

### 2.2. PLA in Natural Fiber Composites

Among natural fibers, flax (F) has a low specific weight, high stiffness, and good thermal and acoustic insulation, which makes it suitable for automotive interior or construction material parts. However, the application of natural fiber composites (NFCs) is still limited due to their mechanical properties, which are, of course, not comparable to those of their synthetic counterparts. To maximize stiffness and try to use these fibers for load-bearing applications, Akonda et al. [27] have proposed the investigation of unidirectional (UD) flax/PLA composites with a FVF = 39%. The introduction of flax fibers has a strong impact on the mechanical properties: compared to pure PLA, these samples exhibit very interesting tensile and flexural properties, whose values are summarized in Table 3. The excellent results can be attributed to the good fiber-matrix adhesion as demonstrated by the SEM images, which show very low porosity (less than 4%) and sparse fiber pull-out. By increasing the fiber volume fraction to 44%, the tensile strength measured by Couture et al. [28] is more than doubled. An increase of almost 69% is observed in flexural strength: this indicates that it is possible to achieve significantly improved mechanical properties even with a low FVF. Despite the numerous papers in the literature dealing with the quasi-static properties of NFCs, only a few of them specifically concentrate on the analysis of fatigue behavior. For this reason, it is worth mentioning the work by Charca et al. [29]. Tensile and fatigue tests are performed on UD flax/PLA composites with a FVF = 36%. For a better comparison, the results of the quasi-static tests are listed in Table 3. Two different methods are used to evaluate the fatigue limit: the first one uses the stabilized temperature, while the second one uses the dissipated energy. In both cases, the same result is obtained: $\sigma_{\infty }$ = 0.43*$\sigma_{ut}$, where $\sigma_{ut}$ corresponds to the ultimate tensile strength (see Table 3). Based on the proposed results, it can be concluded that these composites can compete with UD glass/epoxy laminates [28], even if further testing is required to fully analyze their behavior. Although the FVFs are not so high, the mechanical properties of the flax/PLA composites discussed so far are remarkably good. This is the result of the good interaction between the fiber and the matrix, even without chemical surface treatments.

In addition to flax, other natural fibers deserve attention. In this regard, the works by Siakeng et al. [30] and Rajeshkumar et al. [31] provide an overview of the developed natural fibers/PLA composites. Among all the proposed solutions jute (J) and kenaf (KF) seem to be promising. Considering FVF in the same range of the above-discussed results, lower but equally interesting mechanical properties are found if compared to flax/PLA (see Table 3). However, it is well known that many factors influence the performance of NFCs: fiber extraction process, location of the plant, architecture, curing temperature, production process, and so on. All these aspects make a direct comparison between different fibers quite difficult. With respect to UD, woven fabrics exhibit lower mechanical resistance. As an example, the work by Manral et al. [32] can be considered. They discuss the tensile, flexural, and Izod impact properties of woven flax, jute, and hybrid flax-jute/PLA composites. The comparison shows that flax and hybrid/PLA composites achieve similar results in terms of tensile strength. In all other cases, the combination of the two fiber types is the best solution as it has a better interfacial bonding with the matrix. However, all these results are much lower than those already presented for UD reinforcements. In particular, a reduction of almost 185% is observed in both tensile and flexural strength.

One possible solution to overcome the limitations associated with NFCs is to improve their performance through surface modifications. In this way, they can be made comparable to synthetic composites and meet industrial requirements. Silane, acetylation, and alkali treatments are among the most common: they have already been used in the literature and have shown positive results [33,34,35,36].

## 3. Processing Techniques for PLA-Based Products

Over the last few years, PLA has gained increasing popularity in manufacturing industries, becoming the most widely used raw material in 3D printing, packaging [37,38], and bioplastics applications. As described above, PLA is a biodegradable thermoplastic polymer obtained from renewable resources, primarily corn starch or sugarcane, making it an attractive alternative to traditional petroleum-based plastic raw materials. Once synthesized, PLA can be transformed into various shapes and forms using different techniques tailored to its properties and specific applications [39]. In particular, the versatility of PLA lies in its ease of processing, low shrinkage, and eco-friendliness, making it suitable for a wide range of applications. An overview of the possible processing techniques used to realize PLA-based products is provided below. Hence, the focus is not on the chemical synthesis of PLA but on the different production technologies and methods for PLA items in relation to the applications in which they will be employed.

Some processing techniques refer to blending and compounding to uniformly mix PLA with various fillers before shaping into final products. Depending on the application, the most common fillers include: (i) natural fibers [40], such as hemp, jute, and flax, described in Section 2.2, (ii) mineral fillers, such as talc or calcium carbonate, which can enhance stiffness and reduce cost but might affect impact resistance, (iii) reinforced materials, such as glass or carbon fibers, which can significantly improve tensile strength and toughness but may reduce ductility, and (iv) nanofillers, such as zinc oxide (ZnO) [41], multiwalled carbon nanotubes (MWCNTs) [42], and organically modified montmorillonite (o-MMT) [43], for the functionalization of PLA-based composites suitable for various industrial applications. Some examples are food packaging [37], sensors fabrication [44], and health monitoring devices [45], but also wave-absorbing performance by incorporating graphene (GR) [46]. Moreover, in automotive applications, the effect of the addition of designed additives on main mechanical properties has been investigated [47], in particular, to improve PLA’s toughness and ductility. Regarding additives, a common treatment involves the use of poly(ethylene glycol) (PEG) as a plasticizer in 3D printing, for which its effects on properties of PLA composites have been analyzed [48,49,50].

When combined with fibers, PLA enhances its mechanical properties, obtaining a lightweight composite material suitable for various applications. In particular, several manufacturing techniques can be adopted to realize these fiber-reinforced PLA composites, depending on the use for which they are designed, and they are explained below:

- Filament winding [51]: this process involves winding fibers impregnated with PLA resin onto a mandrel to create composite parts like tanks or structural components;
- Pultrusion [52,53,54]: continuous fibers are pulled through a resin bath and then through a heated die to cure, forming long and continuous composite shapes;
- Hand and spray lay-up processes [55,56]: layers of fiber and PLA resin are alternately placed in a mold. The choice of manual or automated application depends on the production needs;
- Injection molding [57,58]: it is a common method for producing thermoplastic composites. Fibers are mixed with PLA pellets and then injected into a mold under pressure. It allows for complex shapes and mass production;
- Compression molding [59,60]: layers of fiber and PLA are placed in a heated mold under pressure to form composite parts. This method is suitable for higher volume production;
- 3D printing [61,62]: enclosed in the AM techniques, it can be applied to create fiber-reinforced PLA components.

One of the most prominent applications of PLA is in 3D printing, where it is used as a filament for AM processes. More generally, as briefly mentioned in Section 2, AM processes are a class of techniques adopted in several industries for rapid prototyping and fabrication. As opposed to the traditional subtractive production methodologies, such as milling and turning, AM processes manufacture objects and components from computer models, adding one layer on top of the other through different technologies. Among these, the most widely used is fused deposition modeling (FDM) [63,64], which finds application in the above-mentioned 3D printing. The FDM method consists of heating the material filament until it melts and extruding it through a nozzle onto a build surface. The layer-by-layer construction allows for complex geometries and rapid prototyping without the need for specific tooling or molds. For this reason, in addition to its easy use and low-cost regarding materials and maintenance, 3D printing has spread widely over the years, becoming accessible to everyone, including for educational institutions, entertainment purposes, and hobbies. With reference to 3D printing, a recent study has investigated prepreg filament of carbon fiber-reinforced PLA for independent extrusion with 3D printing technology [65], optimizing the impregnation molds.

Continuous filament fabrication and FDM technologies can allow for specific designs and customized components [66], with particular attention to mass customization. This latter, in particular, is a manufacturing technique that combines the flexibility and the development of items tailored to specific customers with low unit cost and high-volume production. In this context, hybrid manufacturing techniques [67], such as combining FDM and injection molding, can be adopted to realize a single personalized part with minimal production costs.

One of the main limitations of the FDM technology is the surface quality of the created objects and their strength. These issues are attributable to the method of layering the thermoplastic materials, which causes deformations and weaknesses [68]. More specifically, since the production process consists of layering also different types of materials, these layers’ formation would affect its resulting performance. The printing parameters—i.e., nozzle diameter, edge width, layer thickness, printing speed, and temperature—strongly affect both the mechanical properties and the microstructure of the final printed products. Therefore, such parameters are investigated in the manufacture of continuous glass fibers reinforced PLA filament through an optimization process [69], highlighting the key role of the impregnation process and the need to enhance the interfacial bonding between fiber and polymer. In particular, the mechanical properties are related to the density of the fill and the number of outer perimeters on which the strength will depend.

A significant influence has also been detected on the orientation of the print surfaces with respect to the coordination system of the device [70,71]. To solve these issues, a topological optimization [72] of the shape of the components for FDM has been deeply investigated. It takes into account the cellulose content in PLA and its influence on defects, highlighting the possibility of achieving the most optimal shape of components. In particular, higher amounts of cellulose lead to material weakening, and the hydrogen bonds of cellulose cause problems in the extrusion itself. Moreover, cellulose in PLA has a negative effect on the environmental resistance of the material, thus contributing to its easier degradation. Hence, in the perspective of future automotive applications, this is not a good feature of the material. On the other hand, in polymer composites with a PLA matrix, cellulose has been used as filler or reinforcement [48,73]. In particular, it is added in different amounts, shapes, and scales—such as cellulose fibers [74], microcrystalline cellulose (MCC) [75], microfibrillated cellulose (MFC) [76], nanocrystalline cellulose (NCC) [77], and nanofibrillated cellulose (CNF) [78,79]. The size of the cellulose, along with its amount, morphology, and structure, determines the different final properties of the composites [49]. Specifically, smaller particles can enhance the mechanical properties, and larger ones contribute to better formability.

It is also important to underline some physical limitations of the PLA material, such as low glass transition temperature, low thermal stability, high brittleness, and low crystallization rate. In particular, these critical issues consequently affect the PLA processing and the working conditions, such as temperature and other manufacturing parameters [80]. In fact, PLA presents a lower melting point than other types of plastic, which may limit its application in high-temperature environments. Anyway, the incorporation of certain fillers can enhance the thermal stability of PLA, allowing it to withstand higher temperatures [81]. More generally, the improvement of PLA properties is accomplished through additives, such as plasticizers and melt strengthening agents [82,83].

As described earlier, another significant technique for manufacturing PLA is injection molding, especially for producing high-volume parts. In this method, PLA pellets are melted and injected into a mold, where they cool and solidify into the desired shape. This technique is widely used for creating everyday items, such as food containers, utensils, and various consumer products. The strength of injection molding relies on its versatility; in fact, it can accommodate complex shapes and provide a smooth surface finish without requiring extensive post-processing. Environmental factors, such as heat exposure and moisture absorption, can significantly affect the performance of the PLA components during their life service. These degradation effects have been investigated on injection-molded PLA for durable applications [84]. In particular, the study showed that PLA resins do not guarantee long-term durability in environments subject to elevated temperature and humidity.

PLA can also be processed through extrusion—a broad production methodology to which FDM also belongs—where it is melted and forced through a die to create continuous profiles, sheets, films, or other shapes [43,82,83]. This method is particularly advantageous for producing packaging materials, such as biodegradable bags or film wraps [38]. In particular, it enables the production of constant-cross-section parts.

Blow molding is a technique for creating hollow objects, like bottles or containers. In this method, a heated tube of material—called parison—is inflated within a mold to form the desired shape. The raw materials typically adopted for blow molding are thermoplastic and glass, and PLA is also explored for being processed through this technique. An advantage that blow-molded PLA provides is that the product is lightweight and suitable for various applications, reducing the amount of material used and, hence, the overall weight. However, this manufacturing technique has not yet been as widely adopted for PLA compared to conventional polymers since PLA can be sensitive to temperature variation.

## 4. Automotive Components Realized Using PLA Composites

**Figure 1:**
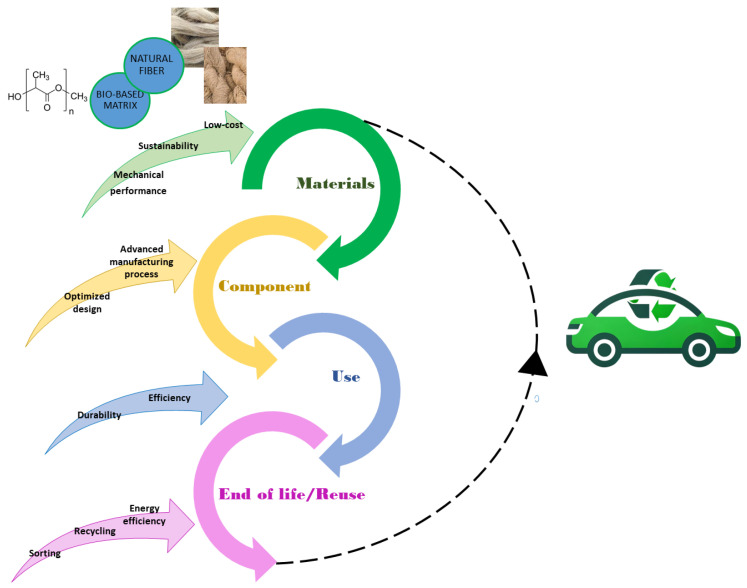
The sustainable lifecycle of a green vehicle within a circular economy.

To address the sustainability and lightweight requirements, the possibility of using PLA is increasingly being affirmed in several applications, including the automotive industry. Evaluating the total emissions from the production, use, and disposal of a vehicle and all its components requires a Life Cycle Assessment (LCA) [85,86,87,88] to be carried out. This comprehensive assessment helps determine the actual sustainability and environmental impact of the materials used. Such evaluations are critical considering the significant emissions produced by traditional vehicles. The US Environmental Protection Agency estimates that a typical passenger vehicle emits about 4.6 metric tons of carbon dioxide (CO_2_) per year [89]. Greenhouse gas (GHG) emissions from traditional vehicles originate not only from the tailpipe but also from the processes involved in gasoline production and distribution. Conversely, electric cars do not produce tailpipe emissions; however, their pollution stems from electricity generation and supply. Therefore, transitioning to green vehicles involves more than just replacing traditional engines with all-electric alternatives [90,91]. So far, from LCA analyses emerged that the mass and size of vehicles significantly affect energy consumption, waste generation, and efficiency. In this sense, it is crucial the use lightweight materials, replacing the traditional low-carbon steel and cast iron, to minimize exhaust during use and improve performance. Their application to vehicle components is extensive yet, from the paneling, such as body panels, seat, and dashboard, to engine or battery, braking system, and structural components, including crash-boxes [92,93,94,95]. In response to the growing need for circularity in industrial processes (see Figure 1), also the selection of sustainable materials is crucial. This choice impacts the initial stages of a lifecycle component, reducing production costs and energy consumption while also minimizing pollution and ensuring renewability.

Lightweight PLA-based composites, for instance, discussed in Section 2, help reduce the environmental impact from raw material extraction to the disposal of components, thanks to their renewable bio-sourced origins.

However, incorporating bio-based materials into existing industrial processes while maintaining the same performance as petroleum-based counterparts is challenging. The manufacturing process, in particular, of bio-based material components can be difficult for several reasons [96,97,98,99]:

- Impurities: impurities in bio-sources can negatively affect the polymerization process and the quality of the final composites.
- Complex chemistry: the chemistry of bio-based materials is more complex than that of petroleum-based materials, making polymerization more challenging.
- Sensitivity to heat and humidity: bio-based materials are highly sensitive to heat and humidity, restricting the temperature and conditions for production and use.
- Degradation over time: bio-based materials tend to degrade over time, which can affect their performance and suitability for polymerization.
- Inconsistency of mechanical properties: natural fibers often exhibit large scattering in mechanical properties due to variations in growing conditions from one crop to another, and their performance is generally poorer compared to synthetic counterparts.

Actually, there is a gap between the conceptualization and the practical implementation of using environmentally friendly materials. Hereinafter, a collection of available literature will be analyzed, prioritizing those who propose the actual use of PLA in automotive components. Additionally, for the sake of completeness, some proposed modeling strategies will also be taken into account. The main design challenge is to be able to satisfy the technical regulations imposed by automotive industry legislation in terms of the structural integrity of the component.

**Figure 2:**
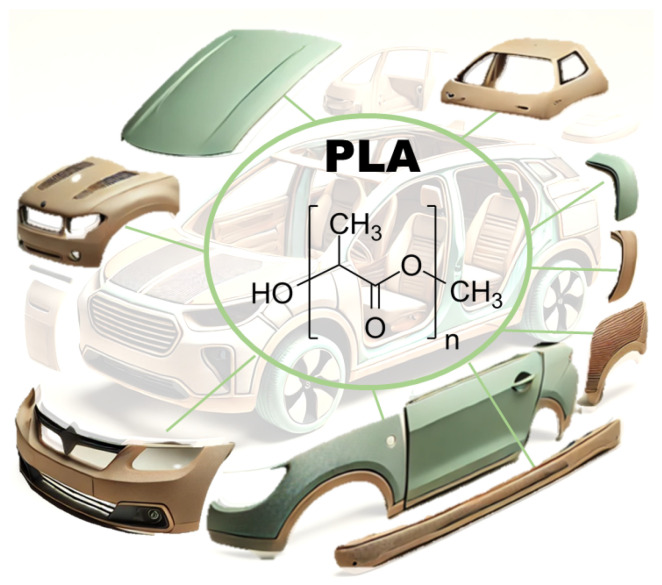
Application of PLA to automotive components: hood, doors, bumper, roof, and side panels.

The use of PLA in automotive applications (see Figure 2) is relatively new. Traditionally, PLA was used, indeed, in packaging applications or as compostable film [100,101,102,103].

The majority of practical implementations of PLA in automotive components involve paneling applications, as demonstrated by the following examples [104]. In fact, ensuring that structural components made from PLA perform comparably to their non-bio-based counterparts remains a significant challenge, mainly due to its inherent low heat resistance and impact strength. In 2016, Mitsubishi Motors with Toray Industries [105], for instance, announced the development of a car floor mat using a combination of PLA and nylon fibers. The high durability of the component was obtained by adding a reforming agent and nylon fibers to increase resistance to light and wear. A 40% reduction in lifecycle CO_2_ emissions and volatile organic compounds (VOCs) of more than 50% over current floor mats was observed.

Bio-based PP/PLA found application in the side trims, door scuff plates, tool box area, floor finishing plate, and package trays of the Lexus CT200h [106]. Kenaf and ramie/PLA have been used in the translucent roof of the Toyota 1/X plug-in hybrid concept vehicle. PLA has also been employed to cover spare wheels on Toyota Prius and Toyota Raum together with kenaf fiber. Moreover, front and rear door scuff plates, cowl side trim board, and rear deck trim cover were made in the Prius model with PLA-PP alloys. Teijin & Mazda developed the Biofront^TM^ stereocomplex PLA and applied it to several automotive components: car seats, floor mats, pillar cover, door trim, front panel, and ceiling material [107]. In Mazda 5 vehicle kenaf/PLA was used in the seat covers, as well as PLA in the interior consoles [108]. Mitsubishi Motors, together with Fiat SpA, have developed interior vehicle components by combining bamboo fibers and a plant-based resin polybutylene succinate. PLA, nylon and flax have been used for floor mats, hemp and cotton for indoor cladding, seat back linings, and floor panels. [10].

Apart from the industry, in recent years, academia has also focused on researching innovative solutions based on the incorporation of PLA as a bio-based alternative to traditional plastics, as reflected in the studies documented in the literature.

Kim et al. [109] proposed the design of a side cover made of jute/PLA composite as an alternative to PP-based materials. Reliability assessment in terms of thermal resistance, light stability, and VOCs was conducted to satisfy the technical requirements. The inherent hydrolysis of PLA and the short time of natural fiber washing were revealed to be decisive for the failure of heat resistance, light resistance, and smell qualification. Mechanical characterization of composites was also performed through tensile, flexural, and impact (Izod) tests. Moreover, thanks to an annealing process, the heat deflection temperature increased by around 250%. An alkali surface treatment of jute was performed aimed at improving the impact properties, which were poorer compared to PP and non-conforming to imposed technical standards, but it was unsuccessful. For this reason, PLA with acrylic rubber compounds was developed, leading to a significant improvement in impact strength. However, effective production for actual implementation as an automotive component was observed to not be possible yet until the prevention of PLA hydrolysis and improved treatment of natural fibers to enhance mechanical performance.

**Figure 3:**
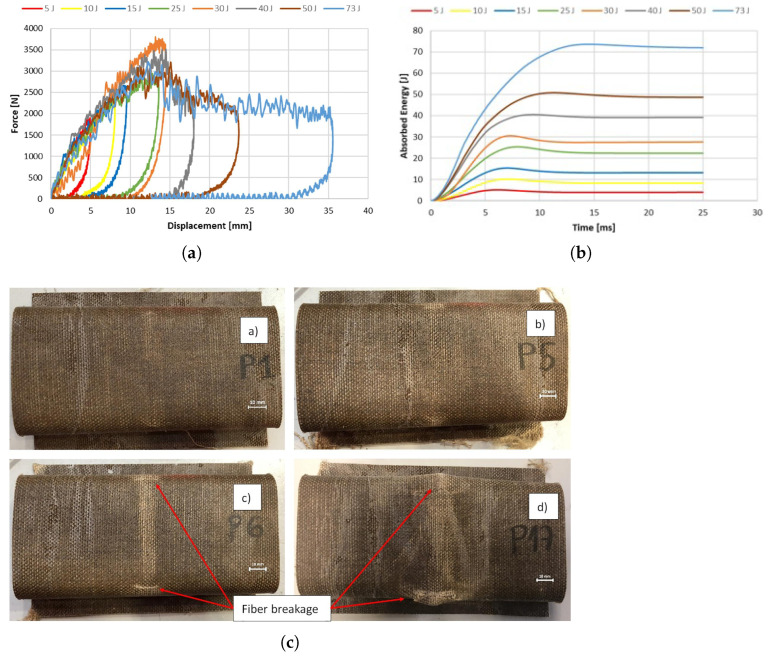
Experimental results of a flax/PLA bumper beam under impact, (**a**) force-displacement, (**b**) energy-time, and (**c**) impacted samples at (a) 5 J, (b) 25 J, (c) 30 J, (d) 73 J [110].

Díaz-Álvarez et al. [110] manufactured flax/PLA bumper beams using through compression molding method, tested under low-velocity impact (LVI), with energies ranging from 5 J to 73 J and bending after impact (BAI). For impact energies between 5 J and 30 J, peak force increased with energy. However, for impact energies from 30 J to 73 J, peak force remained nearly constant at around 350 kN, as demonstrated in Figure 3.

These trends relate to different failure mechanisms: matrix cracking below 30 J and fiber breakage at 30 J or higher. After impact, bending tests showed that the induced damage during impact does not affect residual strength within the applied energy range. This is because bending strength is dominated by fibers oriented longitudinally, while broken fibers are oriented perpendicularly. Below 30 J, residual stiffness was unaffected by impact damage due to matrix cracking. At this level of energy or above, residual stiffness decreases with increasing impact energy. No delamination was observed in any specimen. Future studies should analyze failure mechanisms further to explain why fiber failure at impact energies above 30 J reduces residual stiffness but not residual strength.

**Figure 4:**
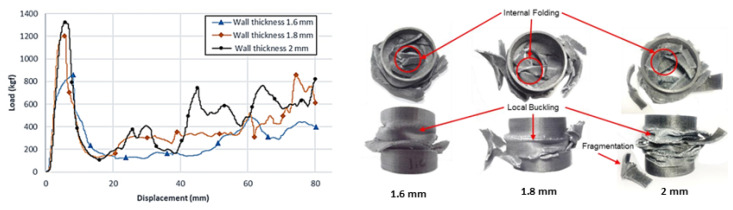
Load-displacement response of carbon/PLA tubes under quasi-static axial compression with the corresponding deformation pattern for wall thicknesses of 1.6, 1.8, and 2 mm [111].

In [111], the crashworthiness of a carbon/PLA circular tube was investigated from an experimental point of view. The component was realized through a 3D printer and tested under axial quasi-static (2 mm/min) compression to evaluate the energy absorption capability. Different wall thicknesses were tested to evaluate the failure mode and the total absorbed energy (AE). The lowest (224 J) and highest (358 J) levels of absorbed energy were reached for the thinnest and thickest tubes, respectively. Unfortunately, the performance of PLA falls significantly short when compared to results [112] that involve the use of carbon fiber and various thermoplastic resins. The mean crush load for carbon-reinforced polyetheretherketone (PEEK), polyethyleneimine (PEI), polyimide (PI), and polyamides (PAs) is about 2 times higher than that of PLA case with comparable thickness for all tested fiber orientations (0°, ±30°). This can be attributed to the different nature of resins. In particular, PEEK guaranteed the best energy absorption value with respect to all mentioned thermoplastic alternatives. Local buckling and brittle fracturing, indeed, emerged as the main deformation mechanism for PLA tubes (see Figure 4), in contrast to the dominant splaying mode.

PEEK exhibits superior mechanical strength and durability. Moreover, it is resistant to fatigue and has high impact strength. In contrast, while PLA is initially strong, it can become brittle over time, especially under load [113,114].

Martin et al. [115] analyzed the effect of incorporating natural fibers (flax, regenerated cellulose, and hemp) and PLA into different automotive components. The mechanical behavior of a footwell panel was evaluated to understand if, under flexure, the maximum deflection could be less than a maximum standard displacement imposed by technical specifications. The hemp fiber provided the highest stiffness to the component, while the one made with cellulose was the least stiff. The flax reinforcement, with properties between those of hemp and cellulose, matched the performance of glass fibers. Overall, test results are satisfactory for passing the standard regulations. Furthermore, glass-filled polypropylene and flax/PLA were used for an automotive structural beam tested through three-point bending. The flax/PLA beams had an average failure load approximately 60% higher than the glass/PP beams. Moreover, a significant difference was observed between the experienced failure modes according to the materials used. For flax/PLA beams, rupture occurred due to the brittle behavior of the material, in contrast to the ductility of glass/PP. On the other hand, the bio-based material configuration proved to be suitable for the prescribed application, as the same stiffness and maximum load as glass/PP could be achieved with less material. Overall, the authors observed that the analyzed bio-based materials could be considered a viable alternative to the non-sustainable benchmarks.

Sopher and Sasaki [116] investigated the bending strength of a composite sandwich panel made of glass fiber-reinforced polymer (GFRP) and a commercial PLA foam (LACTIF®), aiming to produce a prototype of an automotive door panel. From the experimental results, it emerged that by reducing the total weight by up to 50% with respect to traditional sheet molding compound (SMC), glass-reinforced polyester, it is still possible to obtain the same value for the bending strength. Compared to traditional steel door panels, the use of PLA allowed for more lightweight, with about 40% of weight reduction and sustainable solutions. In pursuit of a fully sustainable component, glass fiber could be replaced by natural fibers, at least for the inner panel. Due to the main challenges related to their poor mechanical properties, in fact, particularly in terms of strength and puncture resistance, carbon or glass fibers currently remain the best choice for the outer panel.

In [117], Huvat et al. studied the effect of layer thickness, infill density, and object orientation on the accuracy of 3D printed parts by FDM, as well as on the surface roughness and mechanical strength of PLA. In terms of the length of the component, it emerged that layer thickness was the most significant parameter. For mechanical strength, object orientation had the highest effect on the tensile strength of PLA material. After that, a car backseat Headrest Hanger/Hook was then proposed to be designed based on the optimal solution.

Along with the described experimental studies, it is important to note the attempts in the literature to reproduce the observed or expected experimental behavior from a numerical point of view. Finite Element Analysis (FEA) is a computational technique that is largely used in research and development sectors to understand and possibly predict the behavior of a structure with a particular material configuration under the prescribed physical conditions. During the early design stages of a component, the modeling phase is crucial for optimizing the design and saving both cost and time. For this reason, it is worth mentioning the available simulation results in the literature when PLA properties are included in the material card definition. In [118], a Ford Focus bumper was modeled using solid elements, analyzing the effect of the replacement of PP with PLA. The simulation by ANSYS replicated the pendulum-type test as prescribed by the National Highway Traffic Safety Administration and compared the performance of these thermoplastics to evaluate their energy absorption capability. The stress-strain curve showed that PLA has a higher yield point and absorbs more energy (indicated by the area under the curve) up to that point compared to PP. An FEA was performed using ANSYS to compare the quasi-static impact performance of kenaf/PLA and ABS in [119] for a car door panel application. The PLA-reinforced kenaf fiber composite material demonstrated improved passenger safety and an enhanced end-of-life vehicle recycling factor than ABS-based one. The maximum deformation of this new material was reduced by 28.3% if compared to the ABS. The decrement of displacement value is because the kenaf/PLA composite has higher stiffness and higher tensile strength if compared to the ABS. Having these properties means that kenaf/PLA composite can absorb higher impact force; hence, the deformation is reduced. This also implies that the new material is potentially safer if a side impact occurs due to an accident. Consequently, this bio-polymer composite material was proposed as a promising application in the automotive industry.

**Figure 5:**
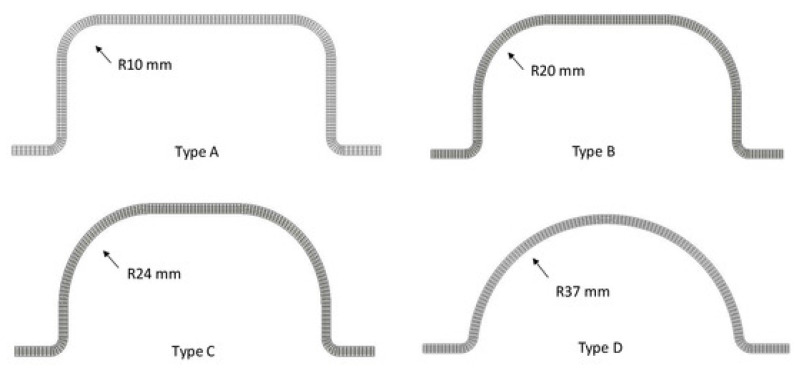
Different cross-sections of bumper beam with the following fillet radii: (A) 10, (B) 20, (C) 24, and (D) 37 mm [120].

Jiao-Wang et al. [120] investigated from a numerical point of view the performance of four different flax/PLA bumpers, according to variation of cross-sectional area, as shown in Figure 5, under low-velocity impact tests with three impact energies (30, 50, 70 J).

**Figure 6:**
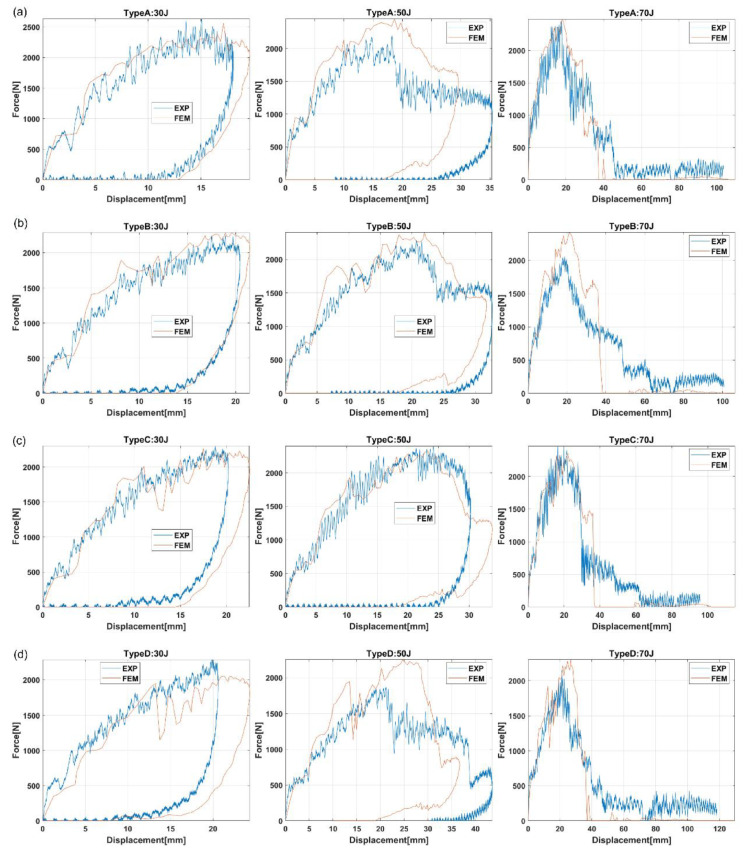
Numerical and experimental comparison of load-displacement responses for all considered energy levels and cross-section geometries: (**a**) Type A, (**b**) Type B, (**c**) Type C, and (**d**) Type D [120].

A bumper model in ABAQUS was defined by four-layer 3D layers, including cohesive contact properties in between to reproduce delamination failure. The results were validated by comparing them with the experimental test outcomes in terms of energy absorption, contact-force history, and extension of delamination. It emerged that simulations accurately predict the experimental response in terms of load-displacement results, energy absorption capability of components under impact (refer to Figure 6), as well as the experienced failure modes. Except for cross-section type D (see Figure 5), an impact energy of around or above 60 J resulted in the highest energy absorption efficiency.

Rounder corners lead to premature delamination, which begins at the section corners and spreads further. The square section (type A) revealed the best geometric configuration for energy absorption. Additionally, the smoother the corners, the lower the peak force observed.

In conclusion, while PLA-based materials could show promising performance compared to traditional non-sustainable alternatives, their long-term properties must be thoroughly validated before real implementation in automotive applications. This validation should occur under various time periods and harsh environmental conditions to ensure they meet the technical requirements of the automotive industry. One of the most significant challenges in applying innovative bio-based materials is their durability, particularly due to their limited resistance to environmental degradation. In this regard, fatigue and creep tests, as well as dimensional stability and heat distortion temperature tests, are crucial to assess the structural durability of the component in the vehicle [98]. On these topics, a limited number of works exist, such as [121,122], even less so for investigations carried out on components to be ultimately installed in vehicles. PLA, in fact, is inherently flammable, and its combustion generates molten material. As an aliphatic polyester, PLA experiences a violent breakdown of its molecular chains at elevated temperatures rather than forming crosslinked structures. To improve flame retardancy and enhance the suitability of this bioplastic for automotive applications, the incorporation of some additives or fillers can be advantageous [123,124], as already discussed in Section 3. Also, bio-based fillers for this objective have been proposed, such as in [125], where a chitosan-based formulation has been employed. Additionally, the use of nanomaterials—such as nanoclays or nanotubes [126]—as well as thermal stabilizers and antioxidants [127], or blending with other polymers [128], are effective solutions for reducing the flammability of PLA.

## 5. Future Developments

The potential for the future application of PLA into the automotive industry passes through the improvement of the polymer and the closer and more effective relation with a larger number of lignocellulosic fibers. As far as the former aspect is concerned, an important breakthrough in the use of PLA for a multiplicity of uses will come when 4D printable shape memory polymers come into mass production, among which PLA is one of the most promising candidates: this can be of interest in the automotive industry whenever fast and reliable actuation is required [129]. For the time being, a number of improvements are actively sought, such as that derived by the application of various blends that confer different properties to the polymer, especially enhancing durability without affecting sustainability, so that at end-of-life chemical recycling can also be proposed [130]. Specific properties can be in particular conferred to PLA by the addition of antioxidants, heat and light stabilizers, and, in particular impact modifiers. In general terms, it can be suggested that the future of PLA would build more on its success as a bio-based polymer, therefore with a lower carbon footprint, and reduce as much as possible the biodegradability during service [62]. A fundamental property for the automotive industry is also the tribological resistance: in this respect, studies on the abrasion performance of PLA have also been proposed and will be likely to diffuse in the future [131]. With respect to the use of different lignocellulosic fibers in the field of PLA matrix natural fiber composites, recent studies do concern both the use of textile reinforcements and molding with short fibers. The use of textiles could be coupled with similar products obtained with PLA, such as those reported by Yang et al., 2021: one of the novel examples is the use of textiles obtained using ramie (Boehmeria nivea), one of the natural fibers with steadily growing market during last years [132]. On the short fibers side, specific innovations concern applications with some novel bio-based fibers, such as pine fibers, duly modified using epoxy to provide suitable interface [133], or rice residues in a potential composite with glass fibers [24]. Once again, the availability of biomass waste or by-products will represent a powerful drive for these developments.

## 6. Conclusions

PLA is the most largely diffused bio-based thermoplastic polymer, especially employed in additive manufacturing processes and often employed in combination with lignocellulosic fibers in natural fiber composites. It is no surprise that also, in view of the increase of the amount of non-petrochemical origin materials in the vehicle, PLA and PLA matrix composites are having an increasingly growing penetration in the automotive sector. To be competitive with other thermoplastics, such as polypropylene, which are of wider use in this field so far, the assessment of mechanical and impact performance is necessary. Especially data on falling weight impact properties are becoming increasingly more available, both on PLA composites reinforced with synthetic fibers and with natural fibers, most particularly flax. These data have the potential to demonstrate the applicability of these materials in structural parts of the car body, such as bumpers, or in other regions of the vehicle, such as doors, where resistance and information over absorbed energy and mode of damage can be considered essential for safety. As a matter of fact, though, the real introduction of PLA as a standard matrix for automotive composites is still mostly based on the application of additive manufacturing processes for their fabrication. Moreover, more rigorous data on fatigue and creep performance of PLA composites are only scantly available so far. This illustrates that the largest parts of the works reported in this review appear rather projections for possible future development of the automotive sector, especially linked with the larger diffusion of 3D and 4D printing in this industry, which is likely to take place in the next few years. Despite these limitations, which include also inherent brittleness and limited thermal stability, the development of PLA composites with a variety of lignocellulosic fibers have been brought forward. Their application in the vehicle, beyond being recommendable in terms of the reduced carbon footprint generated for the production of bio-based polymers, would be likely to flare up in the next decade or so due to improvements in the development of these materials, which are discussed in Section 5.

## Figures and Tables

**Table 1:** Mechanical properties of SCF and CCF/PLA composites [20].

	Tensile Strength [MPa]	Tensile Modulus [GPa]	Tensile Strain [%]	Flexural Strength [MPa]	Flexural Modulus [GPa]
CCF/PLA	245.40	25.94	1.00	168.88	10.63
SCF/PLA	43.75	4.79	2.20	76.33	4.52

**Table 2:** Mechanical properties of carbon/PLA composites [21].

FVF [%]	Tensile Strength [MPa]	Tensile Modulus [GPa]	Flexural Strength [MPa]	Flexural Modulus [GPa]
22	338.58	11.31	256.06	17.85
33	436.94	12.59	259.23	22.86
40	543.80	13.64	309.64	23.18

**Table 3:** Mechanical properties of neat PLA and UD natural fibers/PLA composites.

Material	FVF [%]	Tensile Strength [MPa]	Tensile Modulus [GPa]	Flexural Strength [MPa]	Flexural Modulus [GPa]	Ref.
Neat PLA	-	40.0	3.8	82.0	4.0	[27]
F/PLA	36	234.4	20.6	-	-	[29]
F/PLA	39	151.0	18.5	215.0	18.8	[27]
F/PLA	44	339.0	20.1	363.0	26.0	[28]
J/PLA	50	152.0	5.3	174.0	-	[30]
KF/PLA	35	131.0	15.0	160.0	-	[30]

## Data Availability

No new data were created or analyzed in this study. Data sharing is not applicable to this article.

## Abbreviations

The following abbreviations are used in this manuscript:

ABS	Acrylonitrile Butadiene Styrene
AE	Absorbed Energy
AM	Additive Manufacturing
BAI	Bending After Impact
C	Carbon
CCF	Continuous Carbon Fibers
CNF	Nanofibrillated Cellulose
CO_2_	Carbon Dioxide
F	Flax
FDM	Fused Deposition Modeling
FEA	Finite Element Analysis
FVF	Fiber Volume Fraction
G	Glass
GHG	Greenhouse Gas
GFRP	Glass Fiber-Reinforced Polymer
GR	Graphene
J	Jute
KF	Kenaf
K	Kevlar
LCA	Life Cycle Assessment
LVI	Low-Velocity Impact
MCC	Microcrystalline Cellulose
MFC	Microfibrillated Cellulose
MWCNTs	Multiwalled Carbon Nanotubes
NCC	Nanocrystalline Cellulose
NFCs	Natural Fiber Composites
o-MMT	Organically Modified Montmorillonite
PAs	Polyamides
PE	Poly(ethylene)
PEEK	Polyetheretherketone
PEG	Poly(ethylene glycol)
PEI	Polyethyleneimine
PET	Poly(ethylene terephthalate)
PHAs	Poly(hydroxyalkanoates)
PI	Polyimide
PLA	Poly(lactic acid)
PP	Polypropylene
R	Ramie
SCF	Short Carbon Fibers
SMC	Sheet Molding Compound
TPS	Thermoplastic starches
UD	Unidirectional
VOCs	Volatile Organic Compounds
ZnO	Zinc Oxide

## References

[1] Rahman M.Z., Rahman M., Mahbub T., Ashiquzzaman M., Sagadevan S., Hoque M.E. (2023). Advanced biopolymers for automobile and aviation engineering applications. J. Polym. Res..

[2] Cywar R.M., Rorrer N.A., Hoyt C.B., Beckham G.T., Chen E.Y.X. (2022). Bio-based polymers with performance-advantaged properties. Nat. Rev. Mater..

[3] Babu R.P., O’Connor K., Seeram R. (2013). Current progress on bio-based polymers and their future trends. Prog. Biomater..

[4] Hossain M.T., Shahid M.A., Akter S., Ferdous J., Afroz K., Refat K.R.I., Jamal O.F.M.S.I., Uddin M.N., Samad M.A.B. (2024). Cellulose and starch-based bioplastics: A review of advances and challenges for sustainability. Polym.-Plast. Technol. Mater..

[5] Musa A., Onwualu A. (2024). Potential of lignocellulosic fiber reinforced polymer composites for automobile parts production: Current knowledge, research needs, and future direction. Heliyon.

[6] Kaseem M., Hamad K., Deri F. (2012). Thermoplastic starch blends: A review of recent works. Polym. Sci. Ser. A.

[7] Bouzouita A., Notta-Cuvier D., Raquez J.M., Lauro F., Dubois P. (2017). Poly(lactic acid)-Based Materials for Automotive Applications. Industrial Applications of Poly(Lactic Acid).

[8] Reddy C., Ghai R., Rashmi, Kalia V. (2003). Polyhydroxyalkanoates: An overview. Bioresour. Technol..

[9] Pradeep S.A., Deshpande A.M., Limaye M., Iyer R.K., Kazan H., Li G., Pilla S., Kutz M. (2024). 31-A Perspective on the Evolution of Plastics and Composites in the Automotive Industry. Applied Plastics Engineering Handbook.

[10] Akampumuza O., Wambua P.M., Ahmed A., Li W., Qin X.H. (2017). Review of the applications of biocomposites in the automotive industry. Polym. Compos..

[11] Ferreira F.V., Pinheiro I.F., de Souza S.F., Mei L.H.I., Lona L.M.F. (2019). Polymer Composites Reinforced with Natural Fibers and Nanocellulose in the Automotive Industry: A Short Review. J. Compos. Sci..

[12] Mishra P., Jagadesh T. (2022). Applications and Challenges of 3D Printed Polymer Composites in the Emerging Domain of Automotive and Aerospace: A Converged Review. J. Inst. Eng. (India) Ser. D.

[13] Trivedi A.K., Gupta M., Singh H. (2023). PLA based biocomposites for sustainable products: A review. Adv. Ind. Eng. Polym. Res..

[14] Tawiah B., Yu B., Fei B. (2018). Advances in Flame Retardant Poly(Lactic Acid). Polymers.

[15] Jagadeesh P., Puttegowda M., Boonyasopon P., Rangappa S.M., Khan A., Siengchin S. (2022). Recent developments and challenges in natural fiber composites: A review. Polym. Compos..

[16] Thirugnanasamabandam A., Subramaniyan M., Prabhu B., Ramachandran K. (2024). Development and comprehensive investigation on PLA/carbon fiber reinforced PLA based structurally alternate layered polymer composites. J. Ind. Eng. Chem..

[17] Heidari-Rarani M., Rafiee-Afarani M., Zahedi A. (2019). Mechanical characterization of FDM 3D printing of continuous carbon fiber reinforced PLA composites. Compos. Part Eng..

[18] Ferreira R.T.L., Amatte I.C., Dutra T.A., Bürger D. (2017). Experimental characterization and micrography of 3D printed PLA and PLA reinforced with short carbon fibers. Compos. Part Eng..

[19] Alkabbanie R., Aktas B., Demircan G., Yalcin S. (2024). Short carbon fiber-reinforced PLA composites: Influence of 3D-printing parameters on the mechanical and structural properties. Iran. Polym. J..

[20] Maqsood N., Rimašauskas M. (2021). Characterization of carbon fiber reinforced PLA composites manufactured by fused deposition modeling. Compos. Part Open Access.

[21] Uşun A., Gümrük R. (2021). The mechanical performance of the 3D printed composites produced with continuous carbon fiber reinforced filaments obtained via melt impregnation. Addit. Manuf..

[22] Chen Y., Wei X., Mao J., Zhao M., Liu G. (2024). Experimental analysis of 3D printed continuous carbon/glass hybrid fiber reinforced PLA composites: Revealing synergistic mechanical properties and failure mechanisms. Polym. Compos..

[23] Varsavas S.D., Kaynak C. (2018). Effects of glass fiber reinforcement and thermoplastic elastomer blending on the mechanical performance of polylactide. Compos. Commun..

[24] Sun Y., Zheng Z., Wang Y., Yang B., Wang J., Mu W. (2022). PLA composites reinforced with rice residues or glass fiber—A review of mechanical properties, thermal properties, and biodegradation properties. J. Polym. Res..

[25] Wang G., Zhang D., Wan G., Li B., Zhao G. (2019). Glass fiber reinforced PLA composite with enhanced mechanical properties, thermal behavior, and foaming ability. Polymer.

[26] Yang J., Guo Y., Yao L., Ni Q., Qiu Y. (2018). Effects of Kevlar volume fraction and fabric structures on the mechanical properties of 3D orthogonal woven ramie/Kevlar reinforced poly (lactic acid) composites. J. Ind. Text..

[27] Akonda M., Alimuzzaman S., Shah D.U., Rahman A.M. (2018). Physico-Mechanical, Thermal and Biodegradation Performance of Random Flax/Polylactic Acid and Unidirectional Flax/Polylactic Acid Biocomposites. Fibers.

[28] Couture A., Lebrun G., Laperrière L. (2016). Mechanical properties of polylactic acid (PLA) composites reinforced with unidirectional flax and flax-paper layers. Compos. Struct..

[29] Charca S., Jiao-Wang L., Loya J., Martínez M.A., Santiuste C. (2024). High cycle fatigue life analysis of unidirectional flax/PLA composites through infrared thermography. Compos. Struct..

[30] Siakeng R., Jawaid M., Ariffin H., Sapuan S.M., Asim M., Saba N. (2019). Natural fiber reinforced polylactic acid composites: A review. Polym. Compos..

[31] Rajeshkumar G., Arvindh Seshadri S., Devnani G., Sanjay M., Siengchin S., Prakash Maran J., Al-Dhabi N.A., Karuppiah P., Mariadhas V.A., Sivarajasekar N. (2021). Environment friendly, renewable and sustainable poly lactic acid (PLA) based natural fiber reinforced composites – A comprehensive review. J. Clean. Prod..

[32] Manral A., Ahmad F., Chaudhary V. (2020). Static and dynamic mechanical properties of PLA bio-composite with hybrid reinforcement of flax and jute. Mater. Today Proc..

[33] Bajpai P.K., Singh I., Madaan J. (2014). Development and characterization of PLA-based green composites: A review. J. Thermoplast. Compos. Mater..

[34] Sanivada U.K., Mármol G., Brito F.P., Fangueiro R. (2020). PLA Composites Reinforced with Flax and Jute Fibers—A Review of Recent Trends, Processing Parameters and Mechanical Properties. Polymers.

[35] Yang Y., Wan H., Wang B., Wang B., Chen K., Tan H., Sun C., Zhang Y. (2024). Preparation and properties of bamboo fiber/polylactic acid composite modified with polycarbodiimide. Ind. Crop. Prod..

[36] Fang X., Li Y., Zhao J., Xu J., Li C., Liu J., Liu Y., Guo H. (2022). Improved interfacial performance of bamboo fibers/polylactic acid composites enabled by a self-supplied bio-coupling agent strategy. J. Clean. Prod..

[37] Swetha T.A., Bora A., Mohanrasu K., Balaji P., Raja R., Ponnuchamy K., Muthusamy G., Arun A. (2023). A comprehensive review on polylactic acid (PLA)—Synthesis, processing and application in food packaging. Int. J. Biol. Macromol..

[38] Zibaei R., Ebrahimi B., Rouhi M., Hashami Z., Roshandel Z., Hasanvand S., De Toledo Guimarães J., Goharifar M., Mohammadi R. (2023). Development of packaging based on PLA/POE/SeNPs nanocomposites by blown film extrusion method: Physicochemical, structural, morphological and antioxidant properties. Food Packag. Shelf Life.

[39] Yang Y., Zhang M., Ju Z., Tam P.Y., Hua T., Younas M.W., Kamrul H., Hu H. (2021). Poly(lactic acid) fibers, yarns and fabrics: Manufacturing, properties and applications. Text. Res. J..

[40] Ilyas R., Sapuan S., Harussani M., Hakimi M., Haziq M., Atikah M., Asyraf M., Ishak M., Razman M., Nurazzi N. (2021). Polylactic Acid (PLA) Biocomposite: Processing, Additive Manufacturing and Advanced Applications. Polymers.

[41] Dejene B.K., Gudayu A.D. (2024). Eco-Friendly Packaging Innovations: Integrating Natural Fibers and ZnO Nanofillers in Polylactic Acid (PLA) Based Green Composites–A Review. Polym.-Plast. Technol. Mater..

[42] Vidakis N., Petousis M., Kourinou M., Velidakis E., Mountakis N., Fischer-Griffiths P.E., Grammatikos S., Tzounis L. (2021). Additive manufacturing of multifunctional polylactic acid (PLA)—Multiwalled carbon nanotubes (MWCNTs) nanocomposites. Nanocomposites.

[43] Cailloux J., Hakim R., Santana O., Bou J., Abt T., Sánchez-Soto M., Carrasco F., Maspoch M. (2016). Reactive extrusion: A useful process to manufacture structurally modified PLA/o-MMT composites. Compos. Part Appl. Sci. Manuf..

[44] Abdalla A., Hamzah H., Keattch O., Covill D., Patel B. (2020). Augmentation of conductive pathways in carbon black/PLA 3D-printed electrodes achieved through varying printing parameters. Electrochim. Acta.

[45] Ma X., Hu Q., Dai Y., He P., Zhang X. (2022). Disposable sensors based on biodegradable polylactic acid piezoelectret films and their application in wearable electronics. Sens. Actuators Phys..

[46] Yan T., Ye X., He E., Gao Q., Wang Y., Ye Y., Wu H. (2024). GR-Fe3O4/PLA 3D printing composite materials with excellent microwave absorption properties. J. Alloys Compd..

[47] Notta-Cuvier D., Odent J., Delille R., Murariu M., Lauro F., Raquez J., Bennani B., Dubois P. (2014). Tailoring polylactide (PLA) properties for automotive applications: Effect of addition of designed additives on main mechanical properties. Polym. Test..

[48] Benini K.C.C.D.C., Bomfim A.S.C.D., Voorwald H.J.C. (2023). Cellulose-Reinforced Polylactic Acid Composites for Three-Dimensional Printing Using Polyethylene Glycol as an Additive: A Comprehensive Review. Polymers.

[49] Aumnate C., Soatthiyanon N., Makmoon T., Potiyaraj P. (2021). Polylactic acid/kenaf cellulose biocomposite filaments for melt extrusion based-3D printing. Cellulose.

[50] Moscoso-Sánchez F.J., Alvarad A., Martínez-Chávez L., Hernández-Montelongo R., Escamilla V.V.F., Escamilla G.C. (2019). The effects of henequen cellulose treated with polyethylene glycol on properties of polylactic acid composites. BioResources.

[51] Ma Q., Ge J., Rejab M., Sun B., Ding Y., Nie X., Pang H. (2021). Fabrication of the carbon fiber reinforced plastic (CFRP) cone tube through the laboratory-scale 3-axis winding machine. Mater. Today Proc..

[52] Li P., Ma J., He L., Wu S., Yang P., Zhao L., Zhang Y., Zhu Y., Tan H. (2023). Pultrusion preparation and properties of continuous glass fiber reinforced polylactic acid thermoplastic composites. J. Appl. Polym. Sci..

[53] Linganiso L.Z., Bezerra R., Bhat S., John M., Braeuning R., Anandjiwala R.D. (2014). Pultrusion of flax/poly(lactic acid) commingled yarns and nonwoven fabrics. J. Thermoplast. Compos. Mater..

[54] Memon A., Nakai A. (2013). The Processing Design of Jute Spun Yarn/PLA Braided Composite by Pultrusion Molding. Adv. Mech. Eng..

[55] Milenkovic S., Slavkovic V., Fragassa C., Grujovic N., Palic N., Zivic F. (2021). Effect of the raster orientation on strength of the continuous fiber reinforced PVDF/PLA composites, fabricated by hand-layup and fused deposition modeling. Compos. Struct..

[56] Li Y., Ding Q., Zhao H., Wu T., Zhang M., Zhang Y. (2019). Anisotropic Properties of Polylactic acid–carbon Fiber Composites Prepared by Droplet spray Additive Manufacturing. Materials.

[57] Pappu A., Pickering K.L., Thakur V.K. (2019). Manufacturing and characterization of sustainable hybrid composites using sisal and hemp fibres as reinforcement of poly (lactic acid) via injection moulding. Ind. Crop. Prod..

[58] Anuar H., Zuraida A., Kovacs J.G., Tabi T. (2012). Improvement of Mechanical Properties of Injection-Molded Polylactic Acid–Kenaf Fiber Biocomposite. J. Thermoplast. Compos. Mater..

[59] Baghaei B., Skrifvars M. (2016). Characterisation of polylactic acid biocomposites made from prepregs composed of woven polylactic acid/hemp–Lyocell hybrid yarn fabrics. Compos. Part Appl. Sci. Manuf..

[60] Rubio-López A., Olmedo A., Díaz-Álvarez A., Santiuste C. (2015). Manufacture of compression moulded PLA based biocomposites: A parametric study. Compos. Struct..

[61] Almeida V.H.M., Jesus R.M., Santana G.M., Pereira T.B. (2024). Polylactic Acid Polymer Matrix (Pla) Biocomposites with Plant Fibers for Manufacturing 3D Printing Filaments: A Review. J. Compos. Sci..

[62] Tümer E.H., Erbil H.Y. (2021). Extrusion-Based 3D Printing Applications of PLA Composites: A Review. Coatings.

[63] Reverte J.M., Caminero M., Chacón J.M., García-Plaza E., Núñez P.J., Becar J.P. (2020). Mechanical and Geometric Performance of PLA-Based Polymer Composites Processed by the Fused Filament Fabrication Additive Manufacturing Technique. Materials.

[64] Liu Z., Wang Y., Wu B., Cui C., Guo Y., Yan C. (2019). A critical review of fused deposition modeling 3D printing technology in manufacturing polylactic acid parts. Int. J. Adv. Manuf. Technol..

[65] Wang Q., Zhang Q., Kang Y., Wang Y., Liu J. (2023). An investigation of preparation of continuous carbon fiber reinforced PLA prepreg filament. Compos. Commun..

[66] Ying Z., Wu D., Zhang M., Qiu Y. (2017). Polylactide/basalt fiber composites with tailorable mechanical properties: Effect of surface treatment of fibers and annealing. Compos. Struct..

[67] Gong K., Liu H., Huang C., Jiang Q., Xu H., Cao Z., Fuenmayor E., Major I. (2022). Mass Customization of Polylactic Acid (PLA) Parts via a Hybrid Manufacturing Process. Polymers.

[68] Mushtaq R.T., Iqbal A., Wang Y., Khan A.M., Petra M.I. (2023). Advancing PLA 3D Printing with Laser Polishing: Improving Mechanical Strength, Sustainability, and Surface Quality. Crystals.

[69] Chen K., Yu L., Cui Y., Jia M., Pan K. (2021). Optimization of printing parameters of 3D-printed continuous glass fiber reinforced polylactic acid composites. Thin-Walled Struct..

[70] Khosravani M.R., Berto F., Ayatollahi M.R., Reinicke T. (2022). Characterization of 3D-printed PLA parts with different raster orientations and printing speeds. Sci. Rep..

[71] Buj-Corral I., Domínguez-Fernández A., Durán-Llucià R. (2019). Influence of Print Orientation on Surface Roughness in Fused Deposition Modeling (FDM) Processes. Materials.

[72] Kaščak J., Gašpár Š., Paško J., Husár J., Knapčíková L. (2021). Polylactic Acid and Its Cellulose Based Composite as a Significant Tool for the Production of Optimized Models Modified for Additive Manufacturing. Sustainability.

[73] Matsumoto K., Takemura K., Kitamura R., Katogi H., Tanaka T., Takagi H. (2024). Cellulose nanofiber-introduced continuous-ramie yarn-reinforced polylactic acid filament for 3D printing: Novel fabrication process and mechanical properties. Compos. Part Appl. Sci. Manuf..

[74] Liu H., He H., Peng X., Huang B., Li J. (2019). Three-dimensional printing of poly(lactic acid) bio-based composites with sugarcane bagasse fiber: Effect of printing orientation on tensile performance. Polym. Adv. Technol..

[75] Murphy C.A., Collins M.N. (2018). Microcrystalline cellulose reinforced polylactic acid biocomposite filaments for 3D printing. Polym. Compos..

[76] Molinari G., Gigante V., Fiori S., Aliotta L., Lazzeri A. (2021). Dispersion of Micro Fibrillated Cellulose (MFC) in Poly(lactic acid) (PLA) from Lab-Scale to Semi-Industrial Processing Using Biobased Plasticizers as Dispersing Aids. Chemistry.

[77] Ahmad N.D., Kusmono, Wildan M.W., Herianto. (2023). Preparation and properties of cellulose nanocrystals-reinforced Poly (lactic acid) composite filaments for 3D printing applications. Results Eng..

[78] Agbakoba V.C., Mokhena T.C., Ferg E.E., Hlangothi S.P., John M.J. (2023). PLA bio-nanocomposites reinforced with cellulose nanofibrils (CNFs) for 3D printing applications. Cellulose.

[79] Gauss C., Pickering K.L. (2023). A new method for producing polylactic acid biocomposites for 3D printing with improved tensile and thermo-mechanical performance using grafted nanofibrillated cellulose. Addit. Manuf..

[80] Baptista R., Guedes M., Pereira M., Maurício A., Carrelo H., Cidade T. (2020). On the effect of design and fabrication parameters on mechanical performance of 3D printed PLA scaffolds. Bioprinting.

[81] Barczewski M., Mysiukiewicz O., Andrzejewski J., Matykiewicz D., Skórczewska K., Lewandowski K., Jakubowicz M., Aniśko J., Gapiński B., Sałasińska K. (2023). bioXpul™-technology for manufacturing PLA-based biocomposites with increased thermomechanical stability. Manuf. Lett..

[82] Gigante V., Coltelli M.B., Vannozzi A., Panariello L., Fusco A., Trombi L., Donnarumma G., Danti S., Lazzeri A. (2019). Flat Die Extruded Biocompatible Poly(Lactic Acid) (PLA)/Poly(Butylene Succinate) (PBS) Based Films. Polymers.

[83] Mallegni N., Phuong T., Coltelli M.B., Cinelli P., Lazzeri A. (2018). Poly(lactic acid) (PLA) Based Tear Resistant and Biodegradable Flexible Films by Blown Film Extrusion. Materials.

[84] Harris A.M., Lee E.C. (2010). Heat and humidity performance of injection molded PLA for durable applications. J. Appl. Polym. Sci..

[85] European Commission (2020). Determining the Environmental Impacts of Conventional and Alternatively Fuelled Vehicles through LCA.

[86] Subic A., Schiavone F., Leary M., Manning J. (2010). Comparative Life Cycle Assessment (LCA) of passenger seats and their impact on different vehicle models. Int. J. Veh. Des..

[87] Ridge L. (1998). EUCAR-Automotive LCA Guidelines-Phase 2.

[88] Gibson T. (2000). Life Cycle Assessment of Advanced Materials for Automotive Applications. SAE Trans..

[89] Guide G.V. Greenhouse Gas Emissions from a Typical Passenger Vehicle. https://www.epa.gov/greenvehicles/greenhouse-gas-emissions-typical-passenger-vehicle.

[90] Pero F.D., Delogu M., Pierini M. (2018). Life Cycle Assessment in the automotive sector: A comparative case study of Internal Combustion Engine (ICE) and electric car. Procedia Struct. Integr..

[91] Verma S., Dwivedi G., Verma P. (2022). Life cycle assessment of electric vehicles in comparison to combustion engine vehicles: A review. Mater. Today Proc..

[92] Khan F., Hossain N., Mim J.J., Rahman S.M., Iqbal M.J., Billah M., Chowdhury M.A. (2024). Advances of composite materials in automobile applications–A review. J. Eng. Res..

[93] Jeyaguru S., Thiagamani S.M.K., Rangappa S.M., Siengchin S., Krishnasamy S., Muthukumar C., Rangappa S.M., Doddamani S.M., Siengchin S., Doddamani M. (2023). 8-Lightweight and sustainable materials for automotive applications. Lightweight and Sustainable Composite Materials.

[94] Busarac N., Adamovic D., Grujovic N., Zivic F. (2022). Lightweight Materials for Automobiles. IOP Conf. Ser. Mater. Sci. Eng..

[95] Ahmad H., Markina A.A., Porotnikov M.V., Ahmad F. (2020). A review of carbon fiber materials in automotive industry. IOP Conf. Ser. Mater. Sci. Eng..

[96] AL-Oqla F., Omari M. (2017). Sustainable Biocomposites: Challenges, Potential and Barriers for Development. Green Biocompos. Manuf. Prop..

[97] Wang S., Zhang P., Li Y., Li J., Li X., Yang J., Ji M., Li F., Zhang C. (2023). Recent advances and future challenges of the starch-based bio-composites for engineering applications. Carbohydr. Polym..

[98] Chang B.P., Mohanty A., Misra M. (2020). Studies on durability of sustainable biobased composites: A review. RSC Adv..

[99] Murawski A., Diaz R., Inglesby S., Delabar-Monroe K., Quirino R. (2019). Synthesis of Bio-based Polymer Composites: Fabrication, Fillers, Properties, and Challenges. Polymer Nanocomposites in Biomedical Engineering.

[100] Abdul Malek N.S., Faizuwan M., Khusaimi Z., Bonnia N., Rusop M., Asli N. (2021). Preparation and Characterization of Biodegradable Polylactic Acid (PLA) Film for Food Packaging Application: A Review. J. Phys. Conf. Ser..

[101] De Luca S., Milanese D., Gallichi-Nottiani D., Cavazza A., Sciancalepore C. (2023). Poly(lactic acid) and Its Blends for Packaging Application: A Review. Clean Technol..

[102] Ramezani Dana H., Ebrahimi F. (2023). Synthesis, properties, and applications of polylactic acid-based polymers. Polym. Eng. Sci..

[103] Díaz C.A., Pao H.Y., Kim S. (2016). Film Performance of Poly(lactic acid) Blends for Packaging Applications. J. Appl. Poult. Res..

[104] ADAPT A.P.T. History Of Bioplastics In The Automotive Industry. https://adapt.mx/history-of-bioplastics-in-the-automotive-industry/.

[105] Mitsubishi Motors Develops Plant-Based Green Plastic Floor Mat; Green Car Congress; 2006. https://www.greencarcongress.com/2006/06/mitsubishi_moto.html.

[106] Nadda A., Sharma S., Bhat R., Singh H. (2022). Biopolymers Recent Updates, Challenges and Opportunities.

[107] Rusu D., Boyer S., Lacrampe M.F., Krawczak P. (2012). Bioplastics in automotive applications. Proceedings of the Handbook of Bioplastics and Biocomposites Engineering Applications.

[108] Bledzki A.K., Faruk O., Jaszkiewicz A. (2010). Cars from Renewable Materials. Kompoz. Compos..

[109] Jung J.W., Kim S.H., Kim S.H., Park J.K., Lee W.I. (2011). Research on the development of the properties of PLA composites for automotive interior parts. Compos. Res..

[110] Díaz-Álvarez A., Jiao-Wang L., Feng C., Santiuste C. (2020). Energy absorption and residual bending behavior of biocomposites bumper beams. Compos. Struct..

[111] Bintara R., Choiron M. (2021). Deformation pattern and energy absorption of polylactic acid (PLA) carbon crash box under quasi static loading. IOP Conf. Ser. Mater. Sci. Eng..

[112] Ramakrishna S., Hamada H., Maekawa Z., Sato H. (1995). Energy absorption behavior of carbon-fiber-reinforced thermoplastic composite tubes. J. Thermoplast. Compos. Mater..

[113] MatWeb Overview of Materials for Polyetheretherketone, Unreinforced. https://www.matweb.com/search/datasheet_print.aspx?matguid=2164cacabcde4391a596640d553b2ebe.

[114] MatWeb Overview of Materials for Polylactic Acid (PLA) Biopolymer. https://www.matweb.com/search/DataSheet.aspx?MatGUID=ab96a4c0655c4018a8785ac4031b9278.

[115] Martin R., Giannis S., Mirza S., Hansen K. (2009). Biocomposites in challenging automotive applications. Int. Conf. Compos. Mater..

[116] Sopher S., Sasaki H. (2012). Lightweight Door Panel Made with Bio-Based Composite Material. Proceedings of the SAE International.

[117] Huvat R., Azizul M.A., Sulaiman S. (2021). Characteristics of Abs and Pla Material in 3D Printing for Car Backseat Headrest Hanger/Hook Model. J. Automot. Powertrain Transp. Technol..

[118] Guzman-Baeza M., Urriolagoitia-Sosa G., Martínez-Reyes J., Romero-Ángeles B., Soto-Barrón F., Torres-Yáñez A., Contreras-Mendoza B., Hernández-Patiño O. (2023). Implementation of a Biopolymer in an Automotive Bumper. Social Science Research Network.

[119] Fatchurrohman N., Jun H.X., Muhida R., Adelino M.I. Performance Simulation of Bio-Reinforced Composite Car Door Panel using Finite Element Analysis. Proceedings of the 2021 International Conference on Computer Science and Engineering (IC2SE).

[120] Jiao-Wang L., Loya J.A., Santiuste C. (2022). On the Numerical Modeling of Flax/PLA Bumper Beams. Materials.

[121] Kargar E., Ghasemi-Ghalebahman A. (2023). Experimental investigation on fatigue life and tensile strength of carbon fiber-reinforced PLA composites based on fused deposition modeling. Sci. Rep..

[122] Durante M., Formisano A., Boccarusso L., Langella A., Carrino L. (2017). Creep behaviour of polylactic acid reinforced by woven hemp fabric. Compos. Part Eng..

[123] Yang X.M., Qiu S., Yusuf A., Sun J., Zhai Z., Zhao J., Yin G.Z. (2023). Recent advances in flame retardant and mechanical properties of polylactic acid: A review. Int. J. Biol. Macromol..

[124] Lyon R., Walters R. (2005). Flammability of Automotive Plastics.

[125] Xu Y., Zhang W., Qiu Y., Xu M., Li B., Liu L. (2022). Preparation and mechanism study of a high efficiency bio-based flame retardant for simultaneously enhancing flame retardancy, toughness and crystallization rate of poly (lactic acid). Compos. Part Eng..

[126] Xiao Y., Yang Y., Luo Q., Tang B., Guan J., Tian Q. (2022). Construction of carbon-based flame retardant composite with reinforced and toughened property and its application in polylactic acid. RSC Adv..

[127] Zhao P., Liu Z., Wang X., Pan Y.T., Kuehnert I., Gehde M., Wang D.Y., Leuteritz A. (2018). Renewable vanillin based flame retardant for poly(lactic acid): A way to enhance flame retardancy and toughness simultaneously. RSC Adv..

[128] Kervran M., Shabanian M., Vagner C., Ponçot M., Meier-Haack J., Laoutid F., Gaan S., VAHABI H. (2023). Flame retardancy of sustainable polylactic acid and polyhydroxybutyrate (PLA/PHB) blends. Int. J. Biol. Macromol..

[129] Mehrpouya M., Vahabi H., Janbaz S., Darafsheh A., Mazur T.R., Ramakrishna S. (2021). 4D printing of shape memory polylactic acid (PLA). Polymer.

[130] Tripathi N., Misra M., Mohanty A.K. (2021). Durable Polylactic Acid (PLA)-Based Sustainable Engineered Blends and Biocomposites: Recent Developments, Challenges, and Opportunities. ACS Eng..

[131] Ciofu C., Mazurchevici S., Maldonado-Cortes D., Parás L., Correa D., Nedelcu D. (2019). Tribological behavior of PLA biodegradable materials used in the automotive industry. Int. J. Mod. Manuf. Technol..

[132] Yang X., Fan W., Ge S., Gao X., Wang S., Zhang Y., Foong S.Y., Liew R.K., Lam S.S., Xia C. (2021). Advanced textile technology for fabrication of ramie fiber PLA composites with enhanced mechanical properties. Ind. Crop. Prod..

[133] Zhao X., Li K., Wang Y., Tekinalp H., Larsen G., Rasmussen D., Ginder R.S., Wang L., Gardner D.J., Tajvidi M. (2020). High-Strength Polylactic Acid (PLA) Biocomposites Reinforced by Epoxy-Modified Pine Fibers. ACS Sustain. Chem. Eng..

